# Prospective Randomized Controlled Study Comparing IV Lignocaine Infusion Versus IV Magnesium Infusion for Perioperative Outcomes in Reactive Airway Disease Patients Undergoing Laparoscopic Surgery

**DOI:** 10.7759/cureus.89420

**Published:** 2025-08-05

**Authors:** Chandra Shekhar Singh, Swati Verma, Shailendra Singh, Apurva Agarwal, Anil Kumar Verma

**Affiliations:** 1 Department of Anesthesiology and Critical Care, GSVM (Ganesh Shankar Vidyarthi Memorial) Medical College, Kanpur, IND

**Keywords:** intravenous lignocaine infusion, magnesium sulphate, opioid-sparing effect, postoperative analgesia, reactive airway diseases

## Abstract

Introduction: The goal of perioperative management in reactive airway disease (RAD) patients is to ensure optimal airway stability, maintain adequate oxygenation, and reduce the need for mechanical ventilation while minimizing airway irritation and inflammation. Due to the airway hyperresponsiveness and increased risk of respiratory complications in RAD patients, non-opioid adjuncts that provide both bronchodilation and analgesia are preferred. Lignocaine and magnesium sulfate (MgSO₄) have emerged as effective agents in this context.

Method: A total of 60 patients were randomized into two equal groups: group A (lignocaine, n = 30) and group B (magnesium, n = 30). Drugs were given according to the respective groups before induction of anesthesia until the end of surgery.

Result: The data implied that lignocaine may provide more consistent early analgesia than magnesium. Peak airway pressure was comparable in both groups. Hemodynamics and oxygen saturation (SpO₂) were not significantly different between the groups, and the rescue analgesia requirement was less in the lignocaine group. Concerning sedation score and postoperative nausea and vomiting, there was no significant difference between the groups. The duration of hospital stay in the lignocaine group showed a higher mean duration (4.07 ± 0.37 days) compared to the magnesium group (3.53 ± 0.51 days).

Conclusion: These results suggest that the choice between lignocaine and magnesium can be individualized based on specific clinical goals: lignocaine may be preferred when immediate postoperative analgesia is the priority, whereas magnesium may be favored for promoting better pulmonary recovery and a faster discharge from the hospital.

## Introduction

Reactive airway disease (RAD) is a broad term that encompasses a group of conditions characterized by heightened airway sensitivity to various stimuli, including physical, chemical, and pharmacologic agents [1]. Conditions like asthma and chronic obstructive pulmonary disease (COPD) fall within this category, with patients being at higher risk of perioperative respiratory complications [2,3]. The goal of perioperative management in these patients is to ensure optimal airway stability, maintain adequate oxygenation, and reduce the need for mechanical ventilation while minimizing airway irritation and inflammation [4]. Opioid analgesics used for pain control may cause respiratory depression, which is particularly concerning in RAD patients with pre-existing compromised lung function [5,6]. Given these challenges, alternative anesthetic strategies and adjuncts are required to minimize airway reactivity, enhance perioperative analgesia, and improve overall respiratory function. Due to the airway hyperresponsiveness and increased risk of respiratory complications in RAD patients, non-opioid adjuncts that provide both bronchodilation and analgesia are preferred. Lignocaine and magnesium sulfate (MgSO₄) have emerged as effective agents in this context due to their anti-inflammatory, analgesic, and airway-stabilizing properties [7-10].

## Materials and methods

The study was conducted at the Department of Anaesthesia at GSVM (Ganesh Shankar Vidyarthi Memorial) Medical College, Kanpur, between December 2023 and December 2025. The study was approved by the ethical committee of the institution and was registered in the Clinical Trials Registry - India (Registration number: CTRI/2025/05/087130). All participants provided informed and written consent before being enrolled in the study. The study was designed as a prospective, randomized controlled, double-blinded study, ensuring that participants were randomly assigned to either the intervention or control group by the envelope method. A total of 76 patients were assessed for eligibility. Sixteen patients were excluded (did not meet inclusion criteria or declined to participate), leaving 60 patients who were randomized into two equal groups: group A (lignocaine, n = 30) and group B (magnesium, n = 30).

Inclusion criteria

Patients aged between 18 and 60 years, of either gender, who were diagnosed with RAD and optimized for elective laparoscopic surgery were considered for inclusion. Eligible patients were those with an American Society of Anesthesiologists (ASA) physical status classification of I, II, or III. Only patients scheduled for abdominal laparoscopic surgeries expected to last approximately two hours under general anesthesia were included in the study.

Exclusion criteria

Patients were excluded if they had any known contraindications to intravenous lignocaine or magnesium sulfate, or if they refused to participate in the study. Additional exclusion criteria included severe cognitive impairment, previous participation in the same study, and the presence of significant uncontrolled comorbidities such as diabetes mellitus, cardiovascular disease, or chronic kidney disease. Patients with neuropathic disorders, those with a body mass index (BMI) greater than 30, and individuals on chronic analgesic therapy, particularly steroids, were also excluded from the study.

Group A received injection lignocaine (2%) 1.5 mg/kg intravenously, diluted to 20 mL with normal saline, administered intravenously over 20 minutes as a loading dose, and group B received injection magnesium sulfate (10% w/v) at 30 mg/kg, diluted in 20 mL of normal saline and administered intravenously over 20 minutes as a loading dose. Then, following three minutes of pre-oxygenation with 100% oxygen at 6 L/min, slow induction was carried out using injection of fentanyl 1 mcg/kg, injection of propofol 2 mg/kg, and vecuronium 0.1 mg/kg. Oro-tracheal intubation was performed, and the lungs were ventilated with a gas mixture containing 66% nitrous oxide and 33% oxygen at 2 L/min along with sevoflurane, maintaining a dial concentration of 1% to 0.5%. After induction, continuous drug infusions were initiated according to group allocation and continued intra-operatively until the end of surgery. Group A received an injection of lignocaine (2%) infusion at 1.5 mg/kg/hour IV, prepared in a 50 mL syringe. Group B received an injection of magnesium sulfate (10% w/v) infusion at 10 mg/kg/hour IV, also prepared in a 50 mL syringe.

The effectiveness of each intervention was assessed through a range of intraoperative and postoperative parameters. These included the incidence of bronchospasm during the perioperative period and any changes in airway pressure, particularly peak airway pressure observed during mechanical ventilation. The occurrence of episodes of desaturation, defined as a drop in oxygen saturation (SpO₂) below 95%, was also recorded.

Postoperative pain was evaluated using the visual analog scale (VAS) at predefined time intervals, and the time to first rescue analgesia was documented to assess analgesic effectiveness. Hemodynamic parameters, including heart rate and blood pressure, were continuously monitored both intraoperatively and postoperatively to determine drug safety and cardiovascular stability.

In the postoperative period, the incidence of postoperative nausea and vomiting (PONV) was recorded as an important recovery outcome. Additionally, the condition of the lungs post surgery was assessed through clinical auscultation to evaluate airway recovery. Finally, the total duration of hospital stay was documented as an indicator of overall patient recovery and healthcare resource utilization.

## Results

Demographic details are shown in Table 1. There were no statistically significant differences between groups in terms of sex distribution (χ²(1) = 0.000, p = 1.000), RAD types (χ²(3) = 1.003, p = 0.7932), and age categories (χ²(4) = 7.850, p = 0.097). Similarly, the mean BMI did not differ significantly between groups (t(58) = -0.752, p = 0.455). These findings confirm homogeneity between groups at baseline.

**Table 1:** Baseline demographic and clinical characteristics of the participants. RAD: reactive airway disease; COPD: chronic obstructive pulmonary disease; URTI: upper respiratory tract infection.

Variable	Lignocaine (n = 30)	Magnesium (n = 30)	χ²/t	df	p-value
Sex					
Male	10	10	0.000	1	1.000
Female	20	20			
RAD subtype					
Asthma	3	4	1.003	3	0.7932
COPD	5	7			
URTI	20	17			
Allergic rhinitis	2	2			
Age distribution					
≤20 years	0	1	2.677	3	0.444
31–40 years	3	2			
41–50 years	2	5			
51–60 years	25	22			
BMI (kg/m²)	21.51 ± 2.26	21.99 ± 2.63	-0.75	58	0.455

As shown in Table 2, the intraoperative peak airway pressure between the two groups revealed no statistically significant difference (p = 0.1825). The lignocaine and magnesium groups demonstrated comparable mean peak airway pressures during surgery.

**Table 2:** Intraoperative peak airway pressure between the study groups (cm H₂O).

Group	Mean ± SD (cm H₂O)	t-statistic	p-value
Lignocaine	19.4 ± 1.16	1.363	0.1825
Magnesium	19.03 ± 0.93		

**Figure 1:**
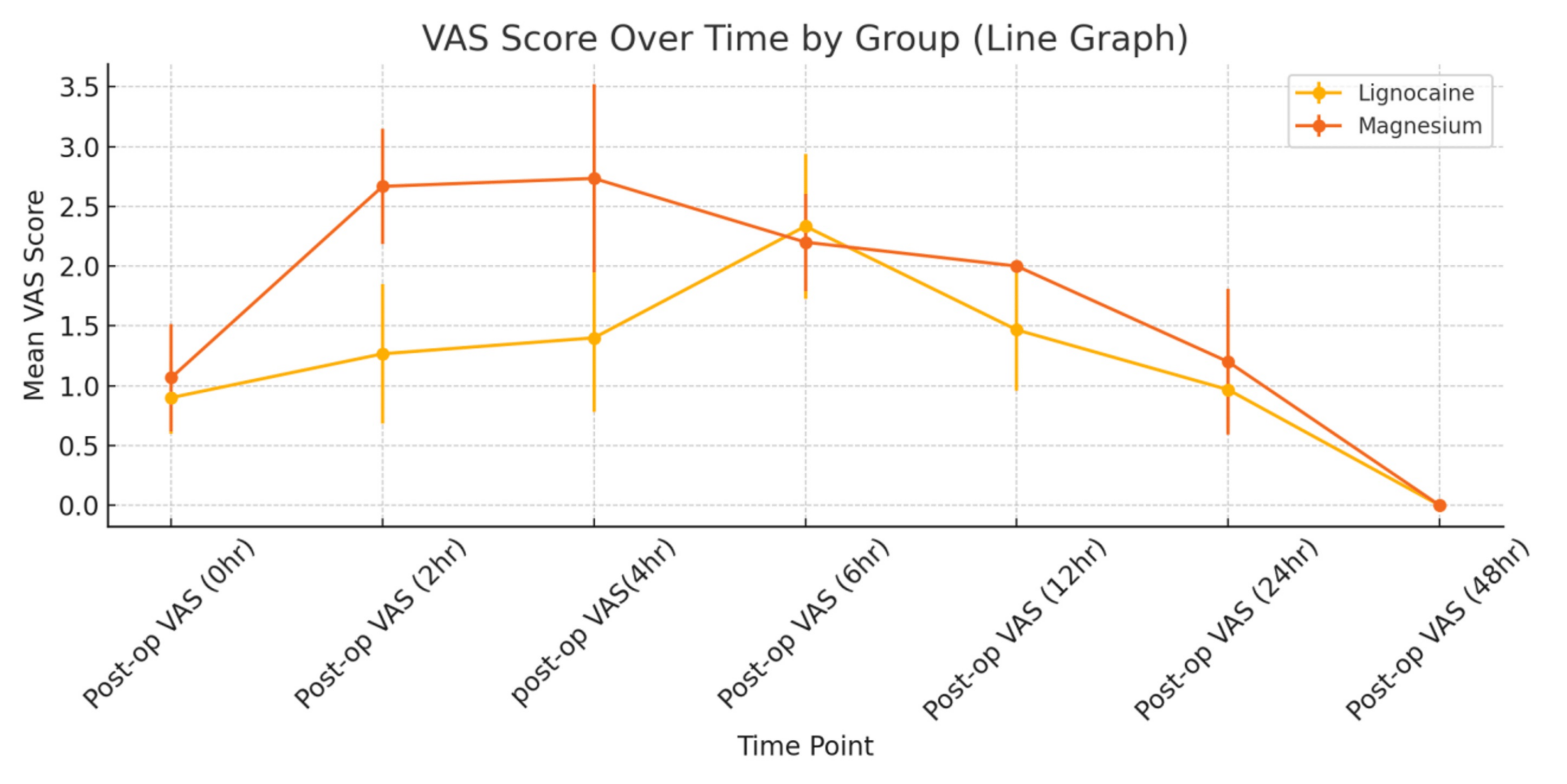
Visual analog scale (VAS) score over time.

Figure 1 shows the mean VAS scores over time for both treatment groups: the magnesium group exhibited significantly higher VAS scores at zero, two, and four hours post operation, suggesting greater pain intensity. The data imply lignocaine may provide more consistent early analgesia than magnesium.

Postoperative sedation scores were recorded at one, two, four, six, 12, and 24 hours using the Ramsay Sedation Score (RSS). At one, two, and four hours, the mean sedation scores were slightly higher in the lignocaine group compared to the magnesium group, but the differences were not statistically significant, with p-values greater than 0.05. At six and 12 hours, both groups demonstrated identical sedation scores (1.17 and 1.03, respectively), with no statistical difference (p = 1.0). By 24 hours, all patients in both groups had returned to a baseline sedation score of 1.00, indicating full recovery from sedative effects. These results suggest that both lignocaine and magnesium provided comparable sedation profiles in the postoperative period, without excessive or prolonged sedation (Table 3).

**Table 3:** Postoperative sedation scores (mean ± SD).

Time point	Lignocaine group	Magnesium group	t-statistic	p-value
1 hour	2.43 ± 0.728	2.30 ± 0.596	0.757	0.4522
2 hours	2.13 ± 0.629	2.23 ± 0.568	-0.646	0.5206
4 hours	2.00 ± 0.743	2.13 ± 0.629	-0.731	0.4675
6 hours	1.17 ± 0.461	1.17 ± 0.379	0.0	1.0
12 hours	1.03 ± 0.183	1.03 ± 0.183	0.0	1.0
24 hours	1.00 ± 0.000	1.00 ± 0.000	-	-

Regarding rescue analgesic requirement, in the lignocaine group, 93.3% of patients did not require any rescue analgesia, while 6.7% required diclofenac. In the magnesium group, 80.0% did not need rescue analgesia, and 20.0% required diclofenac (Table 4).

**Table 4:** Frequency of rescue analgesic use in the postoperative period.

Rescue analgesic		Lignocaine		Magnesium	Test statistics (X^2^)	P-value
None		28 (93.3%)		24 (80.0%)	1.30	0.255
Diclofenac		2 (6.7%)		6 (20.0%)		

There was no statistically significant difference in postoperative SpO₂ levels between the lignocaine and magnesium groups at any time point up to 24 hours (p > 0.05 for all). Suggesting a negligible clinical difference in oxygenation status between the two interventions. This indicates that both intravenous lignocaine and magnesium sulfate infusions were equally effective in maintaining adequate postoperative oxygenation (Table 5).

**Table 5:** Postoperative oxygen saturation (SpO₂) (%) at specified time points.

Time point	Lignocaine group	Magnesium group	t-statistic	p-value
0 hours	98.73 ± 0.694	98.93 ± 0.365	-1.40	0.168
1 hour	98.70 ± 0.651	98.90 ± 0.403	-1.43	0.158
2 hours	98.70 ± 0.596	98.93 ± 0.365	-1.80	0.077
4 hours	98.80 ± 0.607	98.87 ± 0.430	-0.52	0.608
6 hours	98.90 ± 0.305	98.90 ± 0.403	0.00	1.000
12 hours	98.90 ± 0.305	98.93 ± 0.365	-0.35	0.731
24 hours	98.90 ± 0.305	98.97 ± 0.305	-0.89	0.378

The comparison of intraoperative systolic blood pressure readings between the lignocaine and magnesium groups revealed no statistically significant differences at any measured time point (p > 0.05). These findings indicate that both agents are equally effective in maintaining hemodynamic stability during the perioperative period (Table 6).

**Table 6:** Mean systolic blood pressure (mmHg) across different time points.

Time point	Lignocaine (Mean ± SD)	Magnesium (Mean ± SD)	t-statistic	p-value
Preoperative	125.80 ± 9.11	127.70 ± 7.93	-0.86	0.392
5 minutes	130.37 ± 10.73	128.17 ± 11.79	0.76	0.453
10 minutes	131.60 ± 9.75	130.03 ± 13.30	0.52	0.604
15 minutes	129.73 ± 12.23	130.97 ± 11.74	-0.40	0.690
20 minutes	133.17 ± 13.20	129.43 ± 12.50	1.13	0.264
25 minutes	129.83 ± 11.31	131.77 ± 12.01	-0.64	0.522
30 minutes	129.13 ± 11.78	130.33 ± 11.88	-0.39	0.696

There was no statistically significant difference in composite PONV scores between the lignocaine and magnesium groups (p = 0.4298). Both groups demonstrated similar PONV profiles, suggesting that either agent is comparably effective in managing PONV (Table 7).

**Table 7:** Distribution of composite postoperative nausea and vomiting (PONV) scores between the groups.

Group	Score 0.0, No PONV	Score 1.0, Mild nausea	Score 3.0, Vomiting	Test statistics (X^2 ^)	P-value
Lignocaine	22	7	1	1.10	0.4298
Magnesium	21	6	3		

A chi-square test was performed to compare the distribution of postoperative pulmonary status between the lignocaine and magnesium groups. The chi-square value was 1.13, and the p-value was 0.771. Since the p-value is greater than 0.05, the difference is not statistically significant. So the distribution of lung conditions postoperatively appears similar between the two groups (Table 8).

**Table 8:** Group-wise frequency of postoperative lung conditions.

Group		Normal air entry	Reduced air entry	Wheeze		Crepts	Test statistics (X^2^)	P-value
Lignocaine		21 (70.0%)	5 (16.7%)	2 (6.7%)		2 (6.7%)	1.13	0.771
Magnesium		19 (63.3%)	8 (26.7%)	2 (6.7%)		1 (3.3%)		

**Figure 2:**
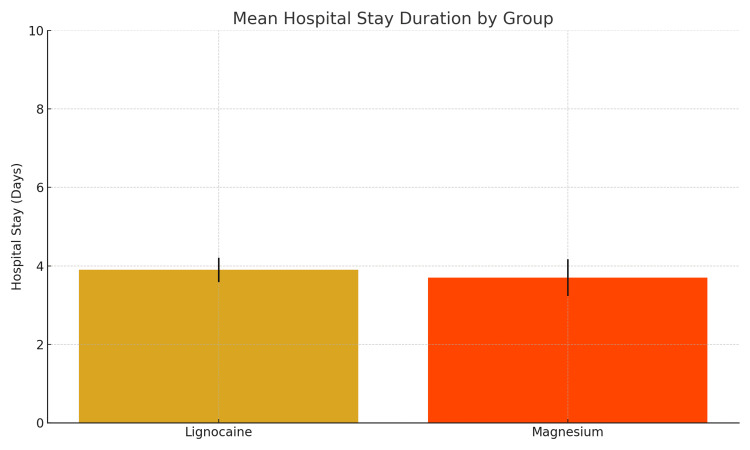
Mean hospital stay duration between the groups (in days).

The duration of hospital stay was assessed in both groups, with the lignocaine group showing a higher mean duration (4.07 ± 0.37 days) compared to the magnesium group (3.53 ± 0.51 days). The difference in hospital stay between the two groups was statistically significant. An independent t-test revealed a t-statistic of 4.67 with a p-value of 0.000018, indicating a significant increase in hospital stay duration in the lignocaine group. This result was further supported by the Mann-Whitney U test (U = 669.0, p = 0.000055), confirming the robustness of the findings even under non-parametric testing assumptions (Figure 2).

## Discussion

Cassuto et al. (2006) [11] reported no adverse reactions to lidocaine in their study of patients post cholecystectomy. These studies highlight the potential benefits of intravenous lidocaine and magnesium in improving postoperative outcomes and reducing opioid use. Koppert et al. (2004) [12] conducted a study on the effects of perioperative IV lidocaine infusion on postoperative pain and morphine consumption after major abdominal surgery, suggesting that lidocaine has opioid-sparing effects and may prevent the development of central sensitization. Ryu et al. (2009) [13] demonstrated that magnesium sulfate reduced analgesic consumption, postoperative pain scores, and shivering incidents, while lowering mean arterial pressure in the immediate postoperative period. A meta-analysis by Marret et al. (2008) [14] reviewed eight trials and concluded that continuous intravenous lidocaine administration during and after abdominal surgery improved rehabilitation and shortened hospital stays. Song et al. (2017) [15] found that intravenous lidocaine infusion reduced cytokine release and improved postoperative recovery by attenuating excessive inflammatory responses following laparoscopic cholecystectomy.

In our study, both lignocaine and magnesium sulfate effectively maintained stable heart rate and blood pressure throughout the intraoperative period. There were no significant intergroup differences in these hemodynamic parameters, underscoring the cardiovascular safety of both drugs in RAD patients undergoing laparoscopy. This finding aligns with existing literature, in which both agents have demonstrated beneficial autonomic modulation without causing significant hypotension or bradycardia.

In terms of analgesic efficacy, intravenous lignocaine provided significantly better early postoperative pain relief than magnesium. Notably, at zero, two, and four hours post surgery, the mean VAS pain scores were significantly lower in the lignocaine group (p < 0.001 for both time points) compared to the magnesium group. However, from six hours onward, pain scores in the two groups became comparable at all subsequent observations (no significant differences at six, 12, 24, or 48 hours postoperatively). Additionally, the requirement for rescue analgesia was low in both groups and did not differ significantly, indicating that although lignocaine offers better pain control in the first few hours after surgery, overall postoperative analgesia was effectively managed by both agents.

Postoperative sedation, measured using the RSS, showed a predictable decline over time in both groups with no significant between-group differences. Both lignocaine and magnesium produced favorable sedation profiles, avoiding excessive sedation or delayed emergence from anesthesia. Similarly, the incidence of PONV was low and statistically comparable between the two groups, further supporting the tolerability of both drugs. These observations suggest that neither lignocaine nor magnesium adversely affected recovery in terms of sedation level or nausea/vomiting, which is important for patient comfort and safety. Respiratory outcomes were of particular interest given the underlying RAD in our patient population. Both groups maintained adequate oxygen saturation levels throughout the postoperative period. However, analysis of postoperative lung auscultation findings revealed a significant advantage in the magnesium group. This improvement is likely attributable to magnesium’s well-known bronchodilatory and anti-inflammatory properties, which can lead to more open airways and fewer adventitious sounds postoperatively. Lignocaine, on the other hand, has airway-stabilizing effects that helped maintain respiratory stability (no cases of severe bronchospasm were noted in our study), but it did not demonstrate the same degree of improvement in auscultation findings as magnesium. Importantly, neither group experienced any major respiratory complications or clinically significant hypoxemia, indicating that both lignocaine and magnesium can be used safely in RAD patients without compromising respiratory function.

One of the most significant findings in this study was the difference in postoperative hospital stay duration between the groups. Patients who received magnesium sulfate had a noticeably shorter mean hospital stay (3.53 ± 0.51 days) compared to those who received lignocaine (4.07 ± 0.37 days). This difference was statistically highly significant (with p < 0.001 on both independent t-test and Mann-Whitney U test analyses). The shorter hospitalization associated with magnesium likely reflects a faster overall recovery, possibly due to fewer subclinical complications or more effective postoperative comfort (including the better pulmonary outcomes noted above). This finding highlights a practical advantage of magnesium in terms of healthcare efficiency and resource utilization, as even a one-day reduction in hospital stay can be meaningful for both patient throughput and cost reduction.

Regarding safety, both drugs exhibited excellent tolerability in our cohort. Only minor and transient adverse events were observed in the lignocaine group (one patient each experienced dizziness and headache), and no significant adverse effects were reported in the magnesium group. Crucially, no patient in either group required an unplanned ICU admission. This favorable safety profile reinforces that both intravenous lignocaine and magnesium sulfate can be administered in RAD patients without a high risk of serious side effects, which is consistent with prior reports of their safety in perioperative use.

## Conclusions

Our findings highlight that each drug has specific advantages. Lignocaine offered an edge in early postoperative pain control, evidenced by significantly lower pain scores in the first hours after surgery. Magnesium sulfate, on the other hand, was associated with better postoperative pulmonary findings (more patients with normal lung auscultation) and a significantly shorter hospital stay, indicating an advantage in terms of pulmonary recovery and overall convalescence. There were no major adverse events or ICU admissions in either group, confirming the excellent safety profile of both interventions. These results suggest that the choice between lignocaine and magnesium can be individualized based on specific clinical goals: lignocaine may be preferred when immediate postoperative analgesia is the priority, whereas magnesium may be favored for promoting better pulmonary recovery and a faster discharge from the hospital.
